# THSD4 is a novel mediator of T cell exclusion and anti-PD-1 resistance in hormone receptor-positive breast cancer

**DOI:** 10.1186/s40364-025-00850-7

**Published:** 2025-10-28

**Authors:** Olivia L. Walker, Marie-Claire D. Wasson, Vishnupriyan Kumar, Sarah Nersesian, Jaganathan Venkatesh, Vishnu V. Vijayan, Lily Coates, Wasundara Fernando, Raj Pranap Arun, Hannah F. Cahill, Elizabeth Baker, Margaret L. Dahn, Drew Slauenwhite, Brianne M. Cruickshank, Penelope Barnes, Modeline N. Longjohn, Thomas James Belbin, Daniel Gaston, Gregory C. Knapp, Jennifer Melvin, Shashi Gujar, Jeanette E. Boudreau, Gillian Bethune, Paola Marcato

**Affiliations:** 1 Department of Pathology, Dalhousie University, Halifax, NS Canada; 2 Beatrice Hunter Cancer Research Institute, Halifax, NS Canada; 3 Department of Microbiology and Immunology, Dalhousie University, Halifax, NS Canada; 4 Biology Department, Acadia University, Wolfville, NS Canada; 5 Nova Scotia Health Authority, Halifax, Canada; 6 Department of Surgery, Dalhousie University, Halifax, NS Canada; 7 Division of BioMedical Sciences, Faculty of Medicine, Memorial University of Newfoundland, St. John’s, NL Canada; 8 Discipline of Oncology, Faculty of Medicine, Memorial University of Newfoundland, St. John’s, NL Canada; 9 Division of Medical Oncology, Department of Medicine, Dalhousie University, Halifax, NS Canada

**Keywords:** THSD4, Breast cancer, Immunotherapy, Biomarker, T cells, Pembrolizumab

## Abstract

Breast cancer remains the most prevalent cancer among women, with hormone receptor-positive (HR +) tumors accounting for approximately 70% of breast cancer cases. While the immune checkpoint inhibitor (ICI) anti-programmed cell death 1 (PD-1) pembrolizumab has demonstrated efficacy in triple-negative breast cancers (TNBCs), its benefit in HR + subtypes is limited. ICI resistance in breast cancer is largely due to a “cold” tumor immune microenvironment characterized by low tumor-infiltrating lymphocytes (TILs). To identify novel genetic determinants of immune exclusion and pembrolizumab resistance, we analyzed multi-omics and clinical datasets from the I-SPY2 clinical trial and The Cancer Genome Atlas (TCGA), focusing on genes associated with low T cell infiltration and poor response to pembrolizumab. We identified thrombospondin type-1 domain containing 4 (THSD4) as a top candidate. *THSD4* expression was significantly elevated in breast tumors with low T cells and in breast cancer patients exhibiting resistance to pembrolizumab, particularly within the HR + subtype. *THSD4* expression is enriched in HR + breast cancers. Validation in local patient cohorts using RNA sequencing and multiplex immunofluorescence confirmed that both high *THSD4* expression and anti-THSD4 antibody staining correlated with reduced T cell infiltration in the tumor epithelium and associations with poorer clinical outcomes. Functional studies in a syngeneic mouse HR + tumor model demonstrated that THSD4 promotes an immunosuppressive tumor microenvironment, with reduced T cells, resistance to anti-PD-1, and altered collagen fiber abundance. Collectively, these findings establish THSD4 as a prognostic biomarker of pembrolizumab resistance and a potential therapeutic target to enhance immunotherapy efficacy in breast cancer.

**Graphical abstract:**

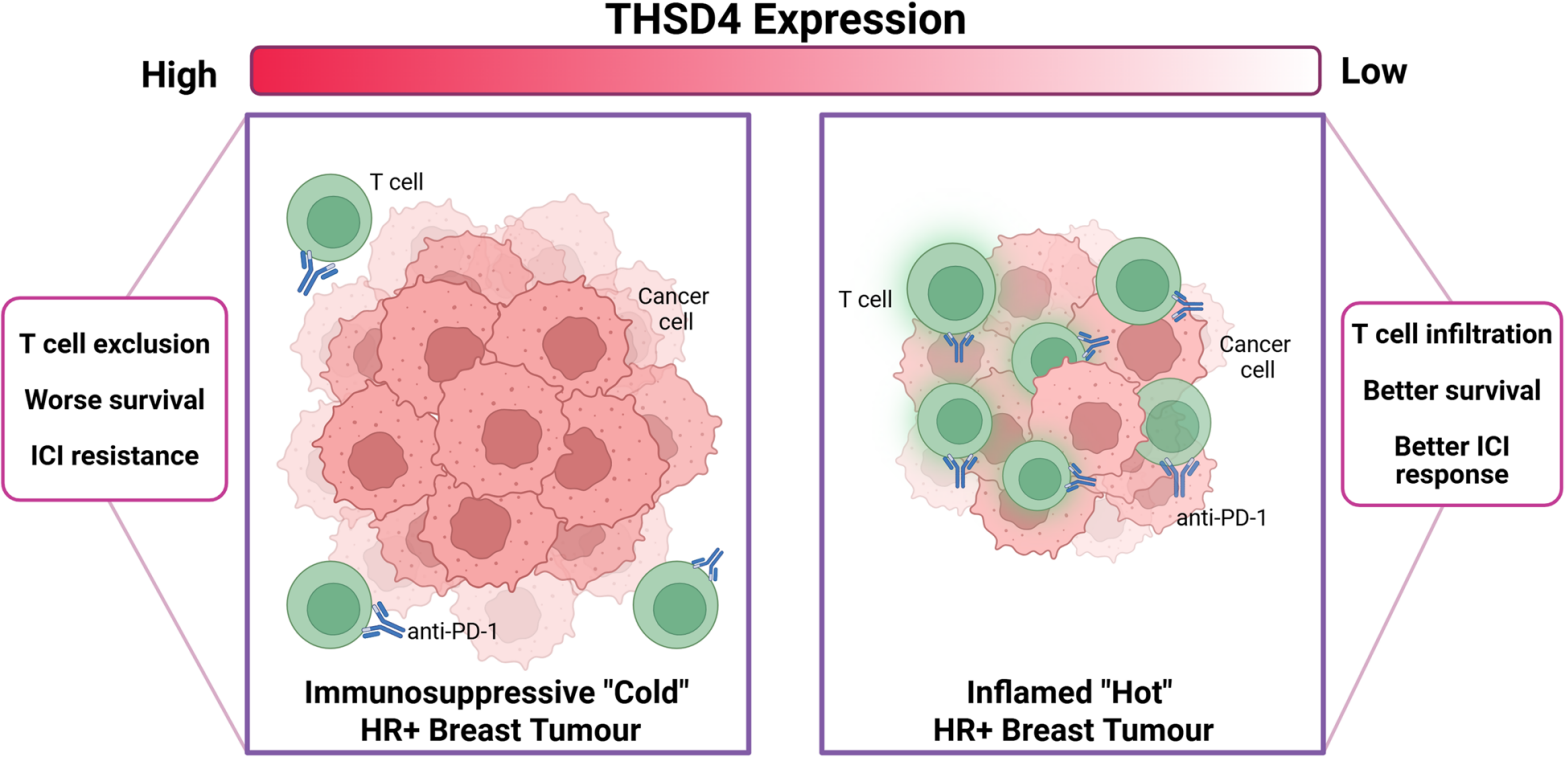

**Supplementary Information:**

The online version contains supplementary material available at 10.1186/s40364-025-00850-7.

To the editor,

Among immune check point inhibitors (ICIs), only the anti-programmed cell death 1 (PD-1) pembrolizumab is approved for use for the triple-negative (TNBC) breast cancer subtype. The efficacy of pembrolizumab in breast cancer, particularly in the hormone receptor-positive (HR +) subtype [1], is limited by incomplete understanding of genetic factors driving immune exclusion and ICI resistance. Identification of novel biomarkers may inform therapeutic strategies to improve response.

We analyzed publicly available datasets to identify genes correlated with both low T cell infiltration and pembrolizumab resistance in breast cancer, identifying thrombospondin type-1 domain containing 4 (THSD4) as a top candidate. Validation using multiplex immunofluorescence of patient cohorts and syngeneic mouse tumor models (methods in Supplementary Material 1) suggests THSD4 is a prognostic biomarker for pembrolizumab resistance and provides first evidence that THSD4 functionally mediates immunosuppression in HR + breast cancers.

## THSD4 is associated with T cell exclusion and pembrolizumab resistance

**Fig. 1:**
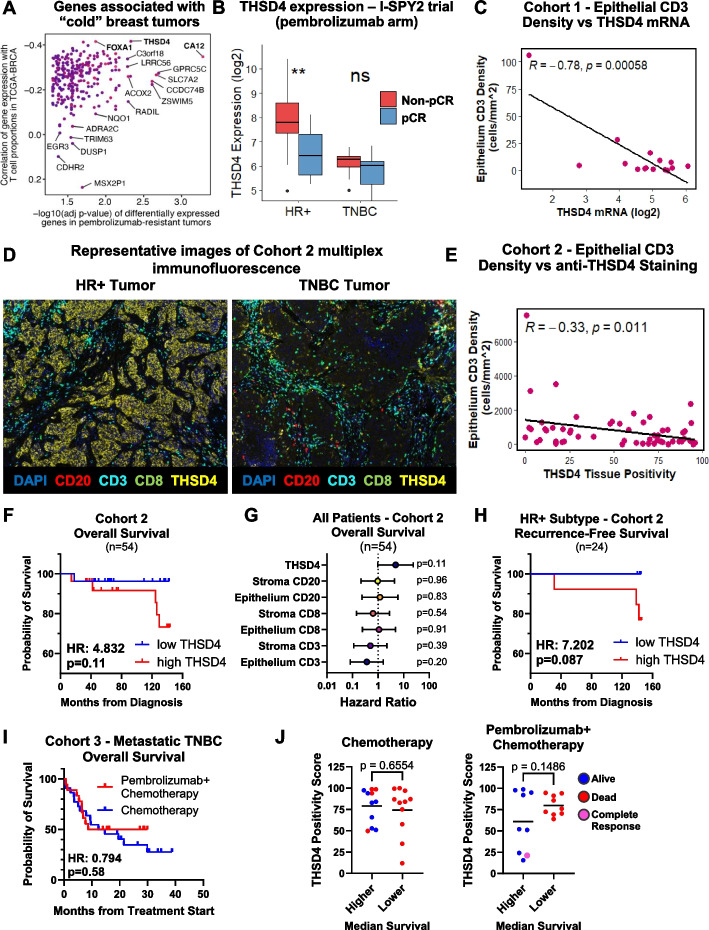
THSD4 is the top gene correlated with pembrolizumab resistance and low T cell infiltration in breast cancer patient datasets and FFPE tumor samples. **A**. Identification of genes most associated with “cold” breast tumors by correlating the genes associated with low T cell proportions in the TCGA-BRCA dataset with genes significantly associated with resistance to pembrolizumab in the I-SPY2 clinical trial. The estimation of tumor composition into the major cell types estimated by CIBERSORTx from bulk single-cell TCGA-BRCA RNA-seq data identified genes significantly associated with low T cell proportions in patient tumours. Pembrolizumab resistance genes were determined using expression of genes in the pre-treatment patient tumors of patients in the pembrolizumab arm compared to the control taxane-anthracycline arm from the I-SPY2 clinical trial that achieved pathological complete response (pCR) versus non-pathological complete response. Significance was based on fold change in gene expression that met the FDR *p*-value cutoffs. The top hits – *THSD4, FOXA1*, and *CA12* – are indicated with bold type. **B**. Comparison of *THSD4* expression in patient tumors from the I-SPY2 clinical trial pembrolizumab arm (*n* = 69) who achieved pathological complete response (pCR, blue) versus those who experienced non-pCR (red) are further sub-grouped based on tumor subtypes, HR + (*n* = 40) or TNBC (*n* = 29). Significance determined by Student’s t-test. *P*-values are represented as: ** *p* < 0.01 and ns = not significant. **C**. Multiplex immunofluorescence of 15 human hormone receptor-positive (HR +) breast tumors stained from Cohort 1 for pan-cytokeratin (panCK), CD20, CD3, and CD45. *THSD4* expression as measured by RNA sequencing (RNAseq). Measured T cell (CD3 + CD8-) density (cells/mm^2^ tissue) in the tumour epithelium plotted versus the log_2_ TPM values of *THSD4* for each tumor. Significance determined by linear model. **D**. Representative images of HR + and TNBC tumors from Cohort 2, consisting of 58 early-stage breast cancer patient tumors, stained by multiplex immunofluorescence staining for CD3 (cyan), CD8 (green), CD20 (red), and THSD4 (yellow). **E**. T cell (CD3 + CD8-) densities per mm^2^ in the tumor epithelium versus anti-THSD4 staining quantified by H-Scoring. **F**. Kaplan–Meier survival plots of all 54 breast cancer patients in Cohort 2 for which complete clinical follow-up data was available based on high versus low anti-THSD4 staining (divided by median positivity H-score) for overall survival from date of diagnosis. HR = hazard ratio. Significance determined by log-rank test. **G**. Forest plot comparing hazard ratio of high compared to low staining of the indicated factors stained by multiplex immunofluorescence of all 54 patients in Cohort 2. **H**. Kaplan–Meier plot as described in **F** for recurrence-free survival, but only for HR + breast cancer patients (*n* = 24). **I**. Kaplan–Meier survival plot of 40 metastatic late-stage TNBC patients from Cohort 3 treated with pembrolizumab + chemotherapy or chemotherapy alone for overall survival from the start of treatment. Hazard ratio (HR) and significance determined by log-rank test. **J**. Comparison of anti-THSD4 H-scoring in the tumor epithelium of patients with lower or higher than median survival in the chemotherapy alone (left) or pembrolizumab + chemotherapy (right) treatment groups. Patients who are alive are indicated by a blue dot, patients who had died are indicated by a red dot, and the patient with complete resolution of disease is indicated by a purple dot. Bar shows mean anti-THSD4 staining. Significance measured by Student’s t-test

Using the transcriptome deconvolution tool CIBERSORTx [2, 3] on TCGA-BRCA RNA-seq data [4–6], we identified genes enriched in “cold” tumors (low T cell infiltration) and cross-referenced these with genes differentially expressed in pembrolizumab-treated early-stage high-risk breast patients from the I-SPY2 trial [1]. *THSD4* is elevated in TCGA tumors with low T cell estimates and in patients resistant to pembrolizumab (Fig. 1A; Supplemental Figure S1 in Supplementary Material 2; Supplementary Material 3 includes all hits).

*THSD4* is higher in HR + breast cancers (Supplemental Figure S2 in Supplementary Material 2). Among the pembrolizumab-treated patients in the I-SPY2 trial, those without pathological complete response (non-pCR) exhibited higher *THSD4*, notably significant in HR + subtypes (Fig. 1B). This was similarly observed in the anti-programmed death-ligand 1 (PD-L1) durvalumab treatment arm [7], suggesting the potential relevance of THSD4 to ICI is not exclusive to pembrolizumab (Supplemental Figure S3 in Supplementary Material 2).

Multiplex immunofluorescence validated these findings in three local cohorts (Supplementary Material 4; Supplemental Figure S4 in Supplementary Material 2). Cohort 1 includes 15 early-stage HR + patients with RNA-seq data; Cohort 2 has 58 early-stage HR + and TNBC patients treated with endocrine or chemotherapy (no ICI); and Cohort 3 comprises 40 metastatic TNBC patients treated with pembrolizumab + chemotherapy or chemotherapy alone based on PD-L1 combined positive score (CPS).

CD3+ T cell staining negatively correlated with *THSD4* mRNA (Fig. 1C, Cohort 1) and anti-THSD4 protein staining in Cohort 2 (Fig. 1D, E). No correlation was seen with CD20 + B cells (Supplemental Figure S5 in Supplementary Material 2). *THSD4* is negatively correlated with CD45 + CD3-CD20- staining in Cohort 1 (Supplemental Figure S6 in Supplementary Material 2); connecting low *THSD4* with non-T and B cell leukocytes. In Cohort 2, high anti-THSD4 staining was associated with poorer overall and recurrence-free survival, especially in HR + patients (Fig. 1F-H; Supplemental Figure S7 in Supplementary Material 2, approaching significance).

In Cohort 3, pembrolizumab + chemotherapy did not improve overall survival compared to chemotherapy alone (Fig. 1I), consistent with known limitations of PD-L1 CPS as a biomarker for pembrolizumab eligibility for late-stage TNBC [8]. Dividing patients by median overall survival revealed similar levels of anti-THSD4 staining among the chemotherapy alone group (Fig. 1J). Pembrolizumab-treated patients with better survival showed lower anti-THSD4 staining approaching significance (Fig. 1J). An exceptional responder’s tumor had among the lowest anti-THSD4 staining. Analysis of other small datasets (less than 20 samples) of metastatic TNBC patients treated with pembrolizumab + chemotherapy similarly failed to associate *THSD4* mRNA with outcomes (Supplemental Figure S8 in Supplementary Material 2). Power analysis of Cohort 3 indicates that a minimum of 62 patients is required to determine if THSD4 staining predicts pembrolizumab response in late-stage TNBC.

## Thsd4 knockdown sensitizes HR + mammary mouse tumors to anti-PD-1 treatment and increases T cell infiltration

**Fig. 2:**
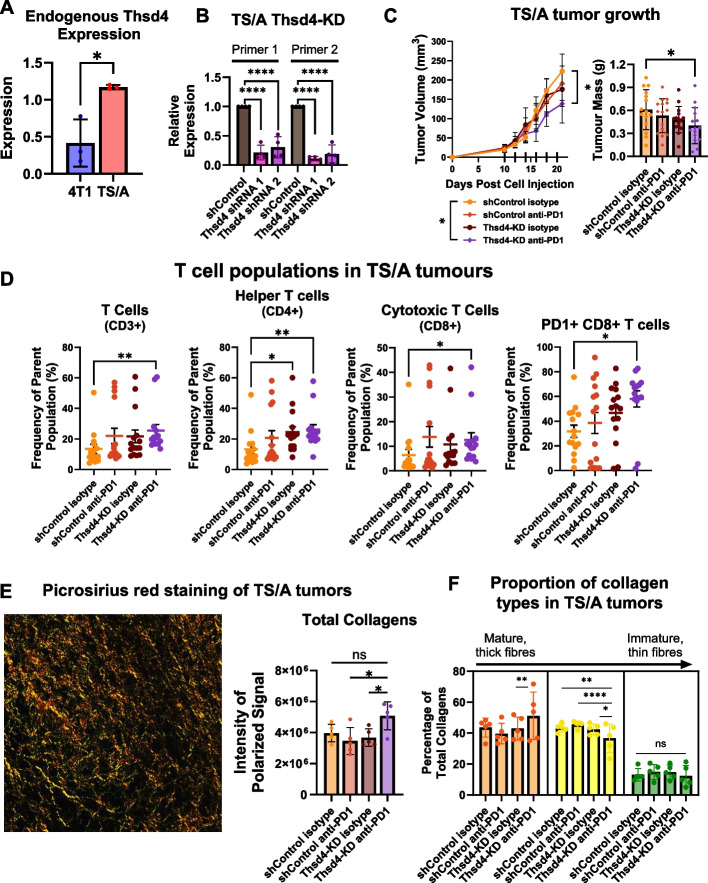
Reducing Thsd4 by knockdown sensitizes HR + TS/A mouse mammary tumours to anti-PD-1 treatment and increases T cell proportions. **A**. Endogenous expression of *Thsd4* comparing the triple-negative 4T1 with the hormone receptor-positive TS/A cell line models by RT-qPCR, compared to reference genes (*n* = 4). **B**. RT-qPCR analysis of TS/A cells harbouring scramble control (shControl) and shRNA targeting *Thsd4* (*n* = 4) with two primers against the gene coding sequence. TS/A cells harbouring scramble control or Thsd4-targeting shRNA 1 were orthotopically implanted into Balb/c mice, once palpable tumours developed they were treated with anti-PD-1 or IgG isotype control to measure tumour growth and immune cell proportions. **C**. Tumour volume (mm^3^, length x width x height / 2) and exponential growth curves (left) and tumour mass (g) (right), mean with SEM error bars, one-way ANOVA. **D**. Flow cytometry analysis of tumours stained for viability, CD45, CD19, CD3, CD4, CD8, PD-1, and PD-L1. T cells are represented as a percentage of the parent population, CD45 + CD19- lymphocytes. The PD-1+ CD8 + T cell parent population are the proportion of CD8 + T cells that are also PD-1 + . Mean with SEM error bars, one-way ANOVA. **E**. TS/A tumours were stained using picrosirius red and visualized under polarized light to measure collagens in the tumours (*n* = 5 / treatment group). The representative image shows the red–orange, yellow, and green collagen fibers against a black background. Total collagen amounts were determined by the mean total polarized signal intensity for three images taken for five tumours from each treatment group. Mean with SD error bars, one-way ANOVA with Tukey’s post-hoc test. **F**. Proportion of tumour collagens that were the red–orange, yellow, or green channel from the polarized signal. Determined by the amount of total polarized signal intensity for the defined colour channels, with the mean of three images taken for five tumours from each treatment group. *P*-values are represented as: * *p* < 0.05, ** *p* < 0.01, *** *p* < 0.001, **** *p* < 0.0001, ns = not significant

To test if THSD4 is functionally relevant, we used two murine breast cancer models: the TNBC 4T1 and HR + TS/A. Consistent with human data, the HR + TS/A line expressed higher Thsd4 than 4T1 (Fig. 2A). Thsd4 knockdown (KD) was achieved in both lines (Fig. 2B; Supplemental Figure S9 in Supplementary Material 2).

In the HR + TS/A model [9], combined Thsd4-KD and anti-PD-1 treatment reduced tumor growth compared to controls (Fig. 2C). No effect was observed in 4T1 tumors, suggesting Thsd4-KD alone cannot overcome innate ICI resistance mechanisms associated with this TNBC model [10] (Supplemental Figure S10 in Supplementary Material 2). The lack of effect in the 4T1 tumors could be due to the endogenous low levels of Thsd4 in 4T1 cells (Fig. 2A). Flow cytometry analysis of the TS/A tumors revealed increased tumor-infiltrating T cells (CD3 +), especially cytotoxic CD8 + and PD-1 + CD8 + subsets, following Thsd4-KD and anti-PD-1 treatment (Fig. 2D; Supplemental Figure S11in Supplementary Material 2), indicating enhanced anti-tumor immunity [11]. Thsd4-KD alone increased CD4 + helper T cells; B cells remained unchanged (Supplemental Figure S11). Although there were no changes in the percentages of CD45 + leukocytes, PD-L1 + CD45 + are increased in the Thsd4-KD and anti-PD-1 treated mice suggesting that other PD-L1 + immune cells could be contributing to the response (Supplemental Figure S11).

As THSD4 belongs to a family of extracellular matrix remodellers [12, 13], THSD4 may affect T cell exclusion by altering the ECM. Picrosirius red staining of TS/A tumors showed that Thsd4-KD plus anti-PD-1 increased total collagen and mature fiber abundance while reducing intermediate fibers (Fig. 2E-F), suggesting changes in ECM composition.

## Conclusion

These data support THSD4 as a functional biomarker of immunosuppression and anti-PD-1 resistance in breast cancer, particularly in HR + subtypes, where it promotes T cell exclusion and modulates ECM structure. Identifying THSD4 offers new potential avenues for therapeutic intervention and patient stratification to enhance pembrolizumab efficacy; however, many questions remain, including evaluating the impact of THSD4 on other immune cell subsets (e.g., myeloid and NK cells). Our results support a causal relationship, as THSD4 knockdown in the HR + TS/A model increased intratumoral T cell numbers and sensitized tumors to anti-PD-1, extending beyond correlative observations in patient samples. Importantly, our analyses addressed potential confounding factors, including tumor purity and stromal proportions, by employing digital cytometry (CIBERSORTx) and multiplex immunofluorescence wherein the association between high THSD4 and reduced T cell infiltration persisted independently of CD20 + B cells.

Regarding T cell dynamics, THSD4 knockdown led to increased CD3 + and CD8 + T cells within tumors, though our current data cannot distinguish whether this reflects enhanced T cell recruitment, proliferation, or survival. Nevertheless, the observed changes, together with ECM remodeling, point to a role for THSD4 in controlling immune access or retention within the tumor microenvironment. Future studies will be required to clarify the precise mechanisms.

A pan-cancer analysis of the ADAMTS-like protein family previously associated THSD4 with tumor tissue upregulation, inflammatory immune subtypes, and transforming growth factor beta (TGF-β) signatures, reinforcing its biological relevance in the tumor microenvironment [14]. Notably, TGF-β signaling is widely recognized as a barrier to effective T cell infiltration and immune surveillance, and spatial profiling studies confirm close interconnections between ECM remodeling, TGF-β pathway activity, and immune exclusion [15].

Looking forward, functional assessments using additional syngeneic and humanized models, as well as THSD4 knockdown and overexpression cell lines across human breast cancer subtypes and patient-derived xenografts, will be critical to clarify the full clinical utility of THSD4 targeting. Investigating THSD4’s effect on ECM degradation, its role in fibroblast biology, and applying proteomics, single-cell RNA-seq, and spatial transcriptomics in low versus high THSD4 tumors may offer deeper mechanistic insight and inform strategies for patient stratification or combination therapy.

## Supplementary Information

Supplementary Material 1. Supplemental Figures and Tables

Supplementary Material 2. Materials and Methods

Supplementary Material 3. All genes hits

Supplementary Material 4. Patient data

## Data Availability

The results shown here are in whole or part based upon data generated by the TCGA Research Network: https://www.cancer.gov/tcga and the I-SPY2 clinical trial (I-SPY2-990 resources. Some of the data presented in this paper were partially funded by the Terry Fox Research Institute’s Marathon of Hope Cancer Centers Network (MOHCCN repository) and the Atlantic Cancer Consortium.

## Abbreviations

CPS: Combined positivity score
ADAMTS: A Disintegrin and Metalloproteinase with Thrombospondin Motifs
ECM: Extracellular matrix
HR: Hazard ratio
HR+: Hormone-receptor positive breast cancer
ICI: Immune checkpoint inhibitor
PD-1: Programmed-death protein 1
PD-L1: Programmed death-ligand 1
TCGA: The Cancer Genome Atlas
THSD4: Thrombospondin type-1 domain containing 4
TIL: Tumor-infiltrating lymphocyte
TNBC: Triple negative breast cancer
