# Supplementary material for: THSD4 is a novel mediator of T cell exclusion and anti-PD-1 resistance in hormone receptor-positive breast cancer
Source: Biomark Res. 2025 Oct 28;13:135. doi: 10.1186/s40364-025-00850-7 (PMC12570572; doi:10.1186/s40364-025-00850-7)
Supplement: Supplementary file 1 — Supplementary Material 1. Supplemental Figures and Tables [file 40364_2025_850_MOESM1_ESM.docx]

**SUPPLEMENTAL FIGURES AND TABLES**


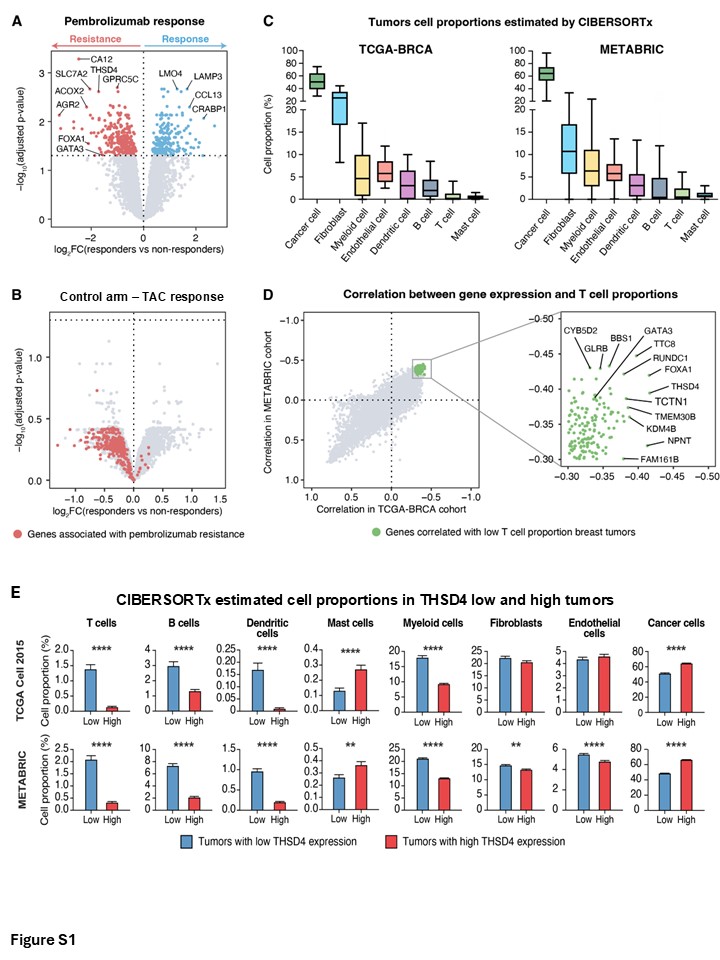


**Supplemental Figure S1. Identification of top genes correlated with pembrolizumab resistance in the I-SPY2 trial and low T cell infiltration by transcriptome deconvolution in breast cancer. A** and **B.** Volcano plots comparing the expression of genes in the pre-treatment patient tumors of patients in the pembrolizumab arm **(A)** or the control taxane-anthracycline arm (**B**) from the I-SPY2 clinical trial that experienced pathological complete response versus non-pathological complete response. Based on fold change in gene expression that meets the FDR p-value cutoffs, genes that are associated with resistance (red) or response (blue) in the pembrolizumab arm. In **B**, no genes meet the FDR cutoff; the genes in **A** that met the FDR cutoff are noted in red in B. **C.** The estimation of tumor composition into the major cell types estimated by CIBERSORTx from bulk single-cell RNA-seq data from the TCGA-BRCA and METABRIC datasets. **D.** Scatter plot demonstrating the identification of genes significantly associated with low T cell proportions in patient tumours and highly correlated between the TCGA-BRCA and METABRIC datasets. The top genes are magnified on the right. **E.** The patients from the TCGA-BRCA (above) and METABRIC (below) were binned into THSD4 low or high expression groups based on based on the top and bottom 33rd percentiles. The proportion of major cell types as estimated by CIBERSORTx between the *THSD4* low and high were compared by Mann-Whiteney test.


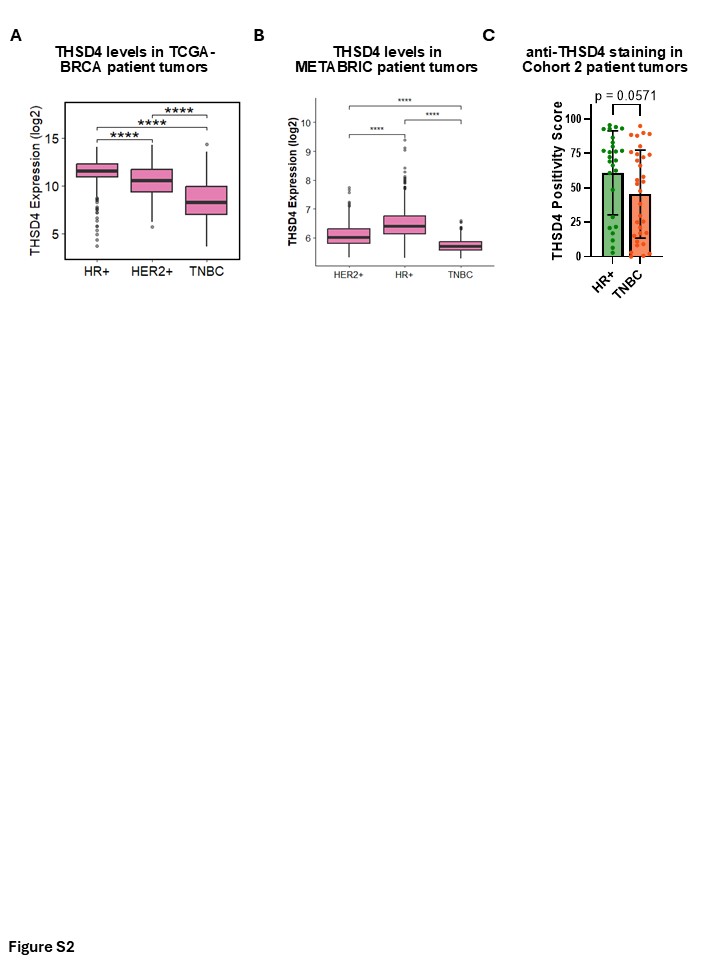


**Supplemental Figure S2. *THSD4* expression is higher in HR+ breast cancers in breast cancer patient tumor datasets and local patient FFPE samples. A.** and **B.** *THSD4* mRNA expression is compared in the hormone receptor positive (HR+), HER-2-enriched (HER2+), triple-negative breast cancer (TNBC) tumors in the TCGA-BRCA (**A**) and METABRIC (**B**) datasets. Significance determined by one-way ANOVA. **C.** Comparison of anti-THSD4 staining as measured by percent positivity of tumor epithelium by H-scoring of multiplex immunofluorescent staining of hormone receptor-positive (HR+) versus triple-negative breast cancer (TNBC) tumors in Cohort 2. Significance determined by Mann-Whitney test.

**
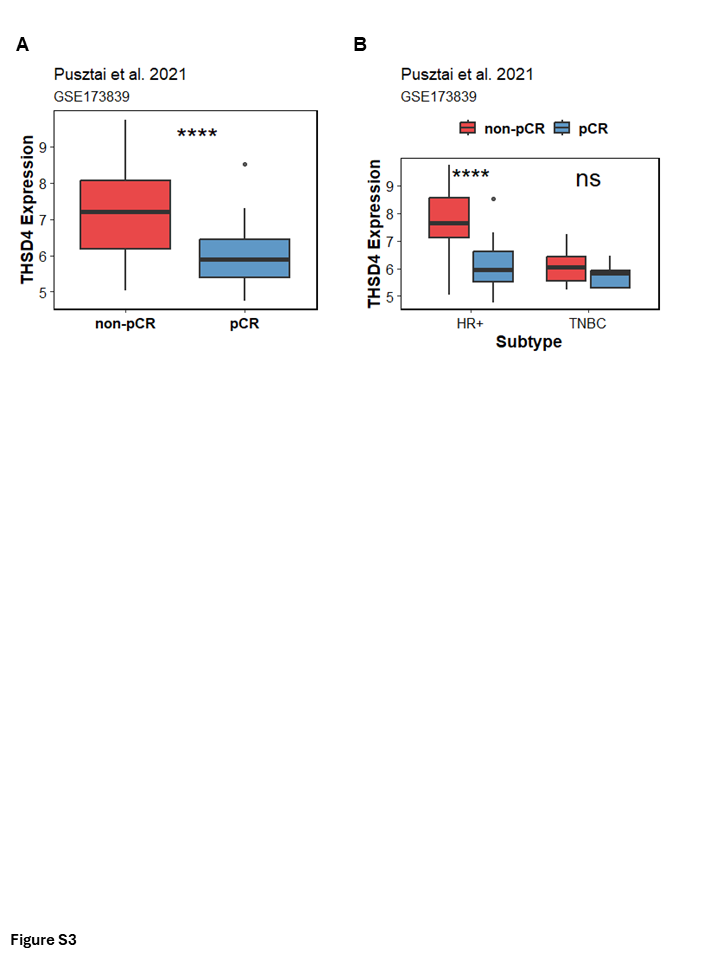
**

**Supplemental Figure S3. *THSD4* *mRNA* expression associated with resistance in the durvalumab/olaparib arm of the I-SPY2 Phase 2 clinical trial in HR+ breast cancers**. **A.** and **B.** *THSD4* mRNA expression in the pre-treatment patient tumors of patients in the durvalumab/olaparib arm of the I-SPY2 clinical trial that experienced pathological complete response (pCR) versus non-pathological complete response were accessed from the Gene Expression Omnibus (GSE173839). The correlation was determined in (**A**) all patients together (n=71) (**B**) and subtyped into hormone receptor positive (HR+; n=50) and triple-negative breast tumors (TNBC; n=21). Significance determined by Student’s T-test


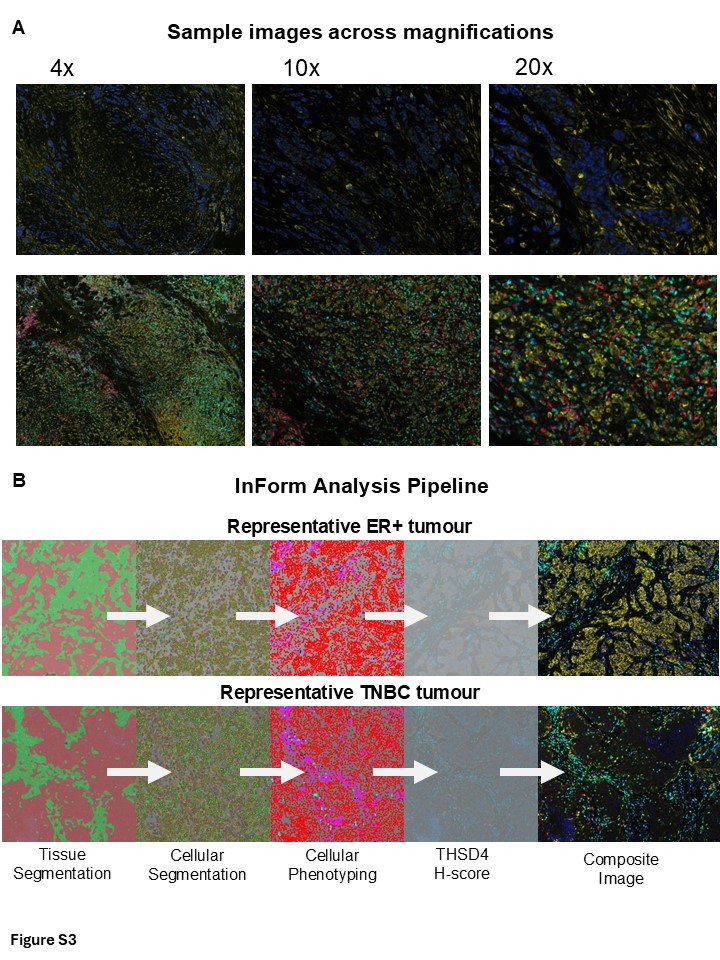


**Supplemental Figure S4. OPAL multiplex immunofluorescence analysis methods. A.** Representative images of breast tumors stained for CD3 (cyan; Opal 480), CD8 (green; Opal 650), CD20 (red; Opal 620), and THSD4 (yellow; Opal 540). Slides were imaged with a 4x, 10x, and 20x objective (above). **B.** Analysis pipeline using InForm software. IM3 images taken on a Mantra workstation are imported into InForm and an algorithm is trained on spectrally unmixed slides (below). Tissues are segmented by manually drawing sections of stroma (red), epithelium (green), or off-core (blue) until adequately refined. Cells are segmented using the nuclear stain DAPI and membrane stains CD3, CD8, and CD20 until adequately refined. Cells are phenotyped based on the expression of CD3 (CD3+ T cells), CD8 (CD3+CD8+ T cells), and CD20 (B cells), or lack of these markers (“other”). H-scoring is completed on the percentage of tumor epithelium that stains positive for THSD4.


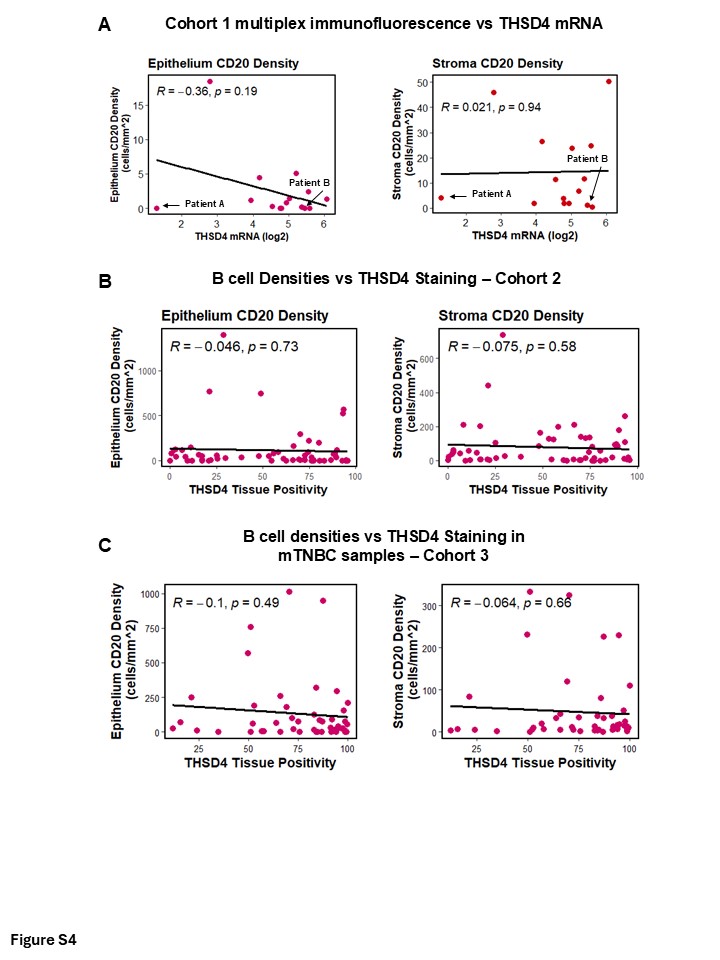


**Supplemental Figure S5. Anti-CD20+ staining is negatively correlated with THSD4 mRNA expression and anti-THSD4 staining.** Three cohorts of Nova Scotian breast cancer patient FFPE samples were stained by Opal multiplex immunofluorescence, comparing B cells (CD20+) to THSD4 expression in the tumor epithelial (left) and stromal (right) compartments. **A.** Fifteen hormone receptor-positive (HR+; Cohort 1) breast cancers were stained by Opal multiplex immunofluorescence for CD45, CD3, CD20, and pan-cytokeratin. THSD4 mRNA was measured by RNA-seq. Correlation between THSD4 mRNA and CD45+CD3-CD20+ cells. **B.** Cohort 2 consisted of 54 early-stage patients (24 HR+, 30 TNBC) and **C.** Cohort 3 consisted of 48 metastatic TNBC patient samples stained for CD20, CD3, CD8, and THSD4. Correlation between CD20+ cells and anti-THSD4 staining. Significance determined by linear model.

**
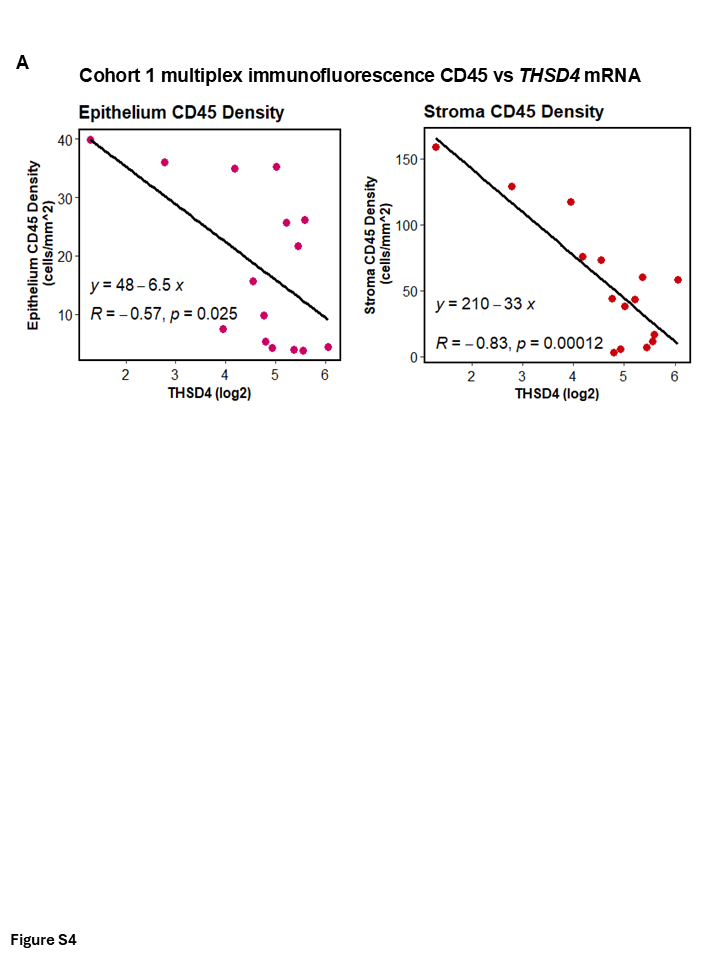
**

**Supplemental Figure S6. Anti-CD45+ staining is negatively correlated with THSD4 mRNA expression.** A cohort of Nova Scotian breast cancer patient FFPE samples were stained by Opal multiplex immunofluorescence, comparing leukocytes (CD45+CD3-CD20-) to THSD4 expression in the tumor epithelial (left) and stromal (right) compartments. Fifteen hormone receptor-positive (HR+; Cohort 1) breast cancers were stained by Opal multiplex immunofluorescence for CD45, CD3, CD20, and pan-cytokeratin. THSD4 mRNA was measured by RNA-seq.


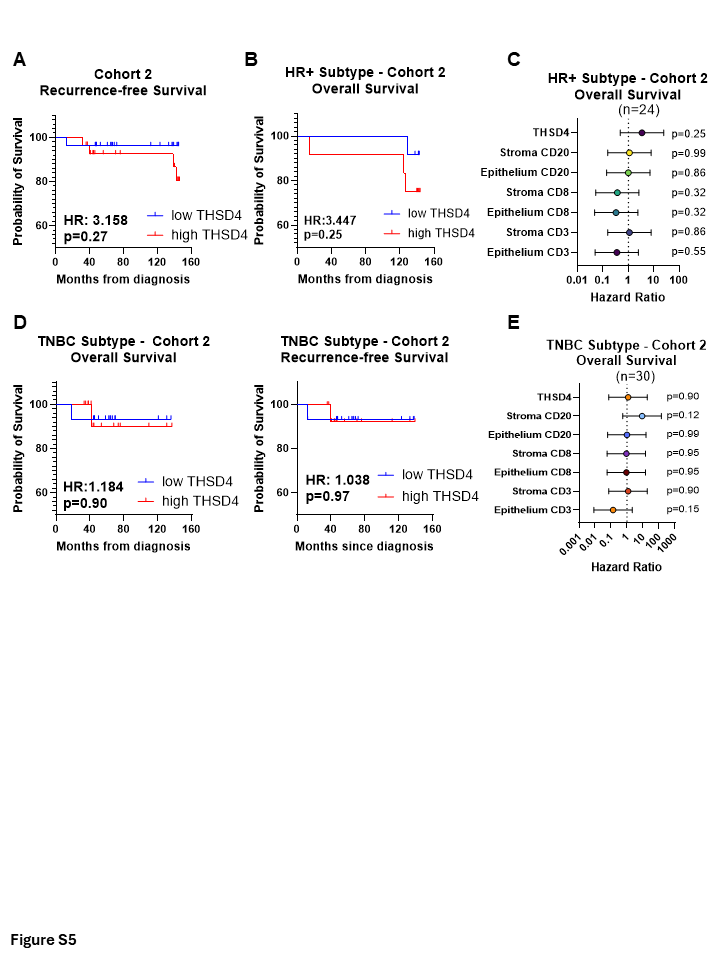


**Supplemental Figure S7. Cohort 2 survival analysis suggests that in HR+ breast cancers anti-THSD4 staining is a better predictor of survival compared to TIL quantification by multiplex immunofluorescence. A.** Kaplan-Meier survival plot of all 54 breast cancer patients in Cohort 2 for which complete clinical follow up data based high versus low anti-THSD4 staining (divided by median positivity H-score) for recurrence-free survival from date of diagnosis. **B.** Kaplan-Meier plot described in **A.** but only for HR+ breast cancer patients (n=24). **C.** Forest plot comparing hazard ratio of high compared to low staining of the indicated factors stained by multiplex immunofluorescence of HR+ patients. **D.** Kaplan-Meier plots described in **A.** but only for TNBC breast cancer patients (n=30) for overall survival (left) and recurrence-free survival (right). **E.** Forest plot comparing described in **C.** of TNBC patients. HR+ = hormone receptor-positive, TNBC = triple negative breast cancer. Significance and hazard ratio (HR) determined by log-rank test.


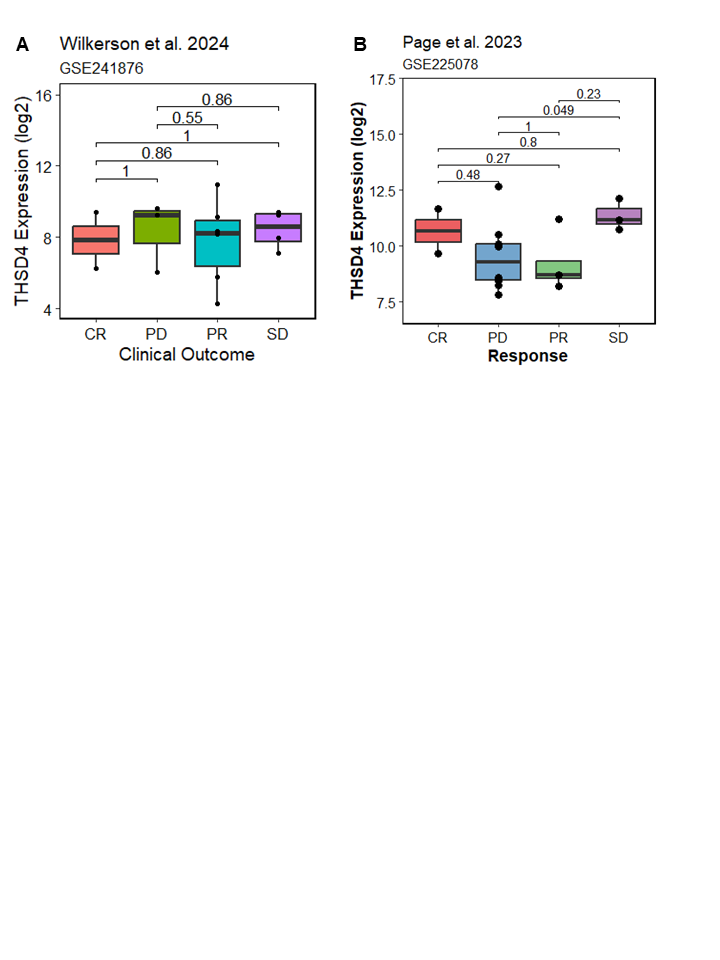


**Supplemental Figure S8. *THSD4* expression is not associated with response in metastatic TNBC patients treated with pembrolizumab and chemotherapy. A.** Normalized RNA-seq counts of *THSD4* expression from pre-treatment biopsies in the tumors from a phase II clinical trial of metastatic TNBCs treated with pembrolizumab + chemotherapy was accessed from GSE241876 and compared with clinical outcome. **B.** Normalized RNA-seq counts of *THSD4* expression from pre-treatment biopsies from a phase Ib clinical trial of metastatic TNBCs treated with pembrolizumab + chemotherapy was accessed from GSE225078 and compared with clinical outcome. Significance determined by one-way ANOVA. CR = complete response, PD = progressive disease, PR = partial response, SD = stable disease.


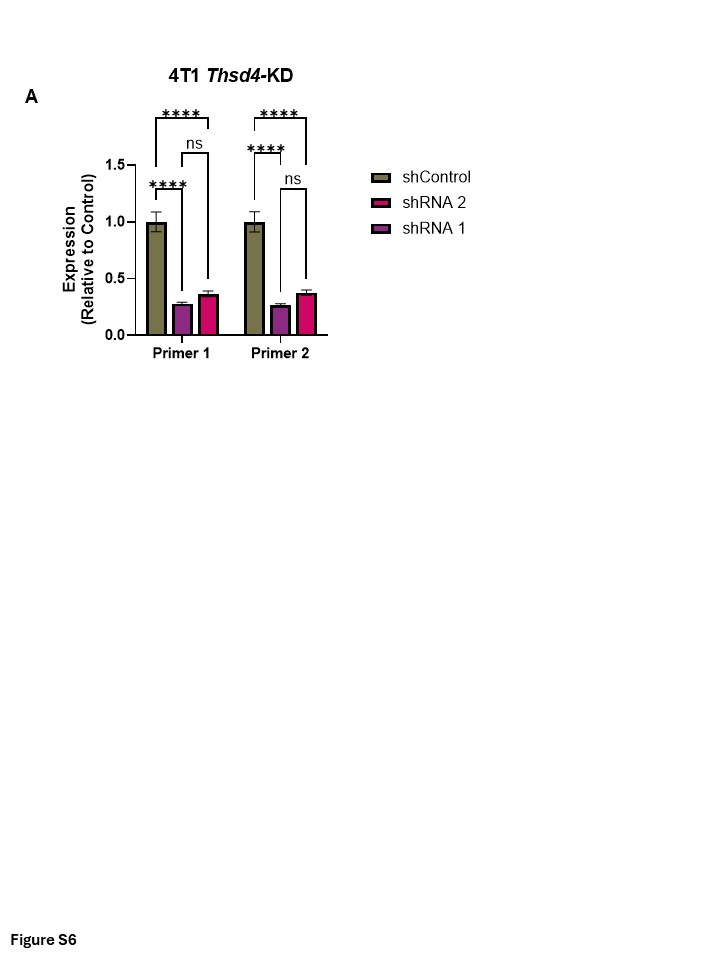


**Supplemental Figure S9. *Thsd4* knockdown validation in 4T1 murine mammary carcinoma model. A.** 4T1 cells harboring shRNA targeting mouse *Thsd4* were validated using two primers by RT-qPCR. Expression was compared to empty vector scramble control (shControl).


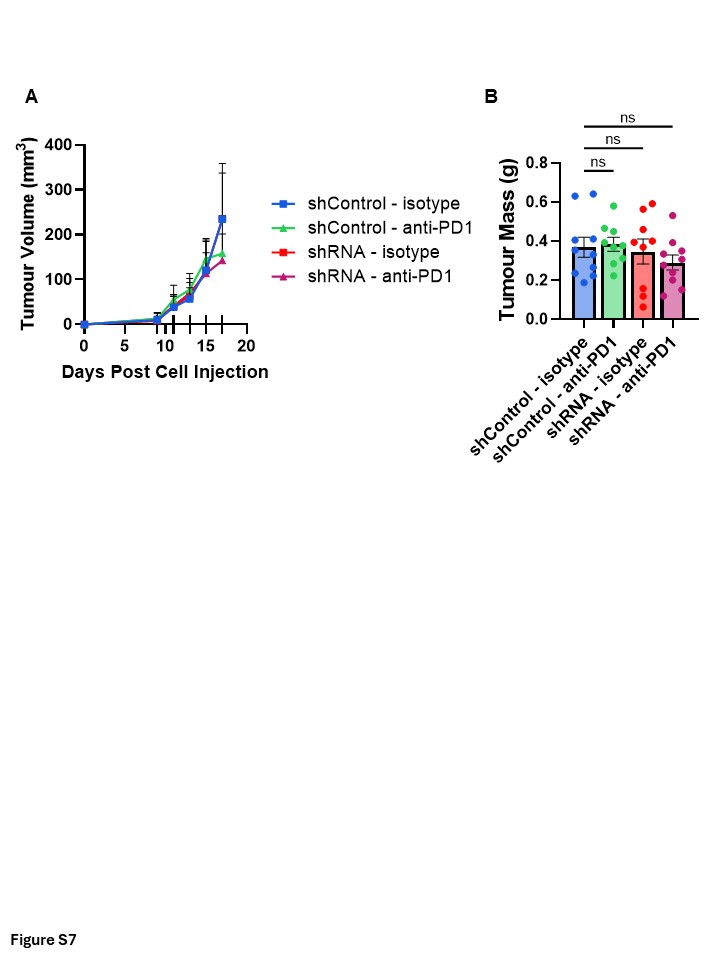


**Supplemental Figure S10. Neither Thsd4 knockdown nor anti-PD-1 treatment decreased 4T1 tumor size.** The murine mammary carcinoma model 4T1 harboring shRNA targeting *Thsd4* were implanted into 7–8-week-old Balb/c mice and treated every other day with anti-PD-1 or isotype control monoclonal antibodies once palpable tumors developed. **A.** Tumor volumes were measured from day 0 until humane endpoint at day 18 (measured as (length x width x height)/2). **B.** Excised tumors were weighed. Significance determined by one-way ANOVA.

**
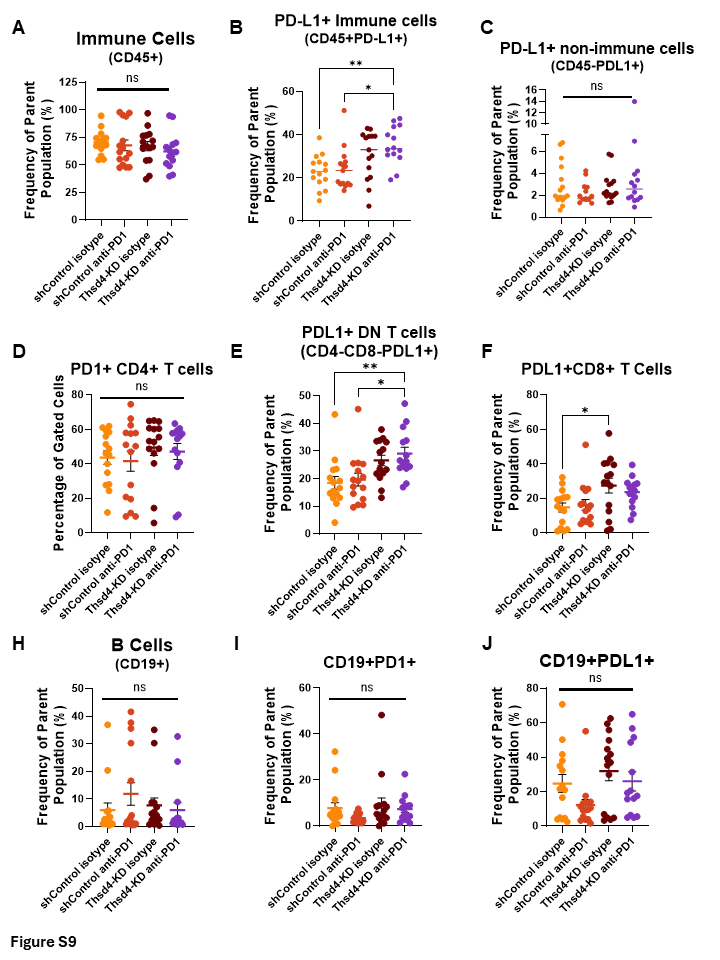
**

**Supplemental Figure S11. Reducing Thsd4 sensitizes HR+ mouse tumours to anti-PD1 treatment and increases PD-L1+ immune cell proportions in HR+ tumours.** Hormone receptor-positive TS/A cells harboring scramble control (shControl) and shRNA targeting *Thsd4* were orthotopically implanted into Balb/c mice, once palpable tumours developed, they were treated with anti-PD1 or IgG isotype control to measure tumour growth and immune cell proportions. Flow cytometry analysis of tumours stained for viability, CD45, CD19, CD3, CD4, CD8, PD-1, and PD-L1. Cells are represented as a percentage of the parent population. Mean with SEM error bars, one-way ANOVA. * p<0.05, ** p<0.01, *** p <0.001.

**Supplemental Table S1.** **pLKO.1 IDs for *Thsd4* shRNA knockdowns in murine mammary carcinoma cells**

| Clone ID | shRNA |
| --- | --- |
| TRCN00000080439 | shRNA 1 |
| TRCN00000080442 | shRNA 2 |

**Supplemental Table S2.** **Primer sequences for RT-qPCR on murine mammary carcinoma cells**

| Gene | Forward Primer | Reverse Primer |
| --- | --- | --- |
| B2m | ATGGGAAGCCGAACATACTG | CAGTCTCAGTGGGGGTGAAT |
| Gapdh | TGGCAAAGTGGAGATTGTTGCC | AAGATGGTGATGGGCTTCCCG |
| Thsd4 primer #1 | CCGAGACGTGAAGTGTGTGA | TCATTTGGCCGGAGCTTCAT |
| Thsd4 primer #2 | CTGTGGGAAAGGATCGCAGT | ACAGTAGCTCTCGGGAACCT |
