# Supplementary material for: THSD4 is a novel mediator of T cell exclusion and anti-PD-1 resistance in hormone receptor-positive breast cancer
Source: Biomark Res. 2025 Oct 28;13:135. doi: 10.1186/s40364-025-00850-7 (PMC12570572; doi:10.1186/s40364-025-00850-7)
Supplement: Supplementary file 2 — Supplementary Material 2. Materials and Methods [file 40364_2025_850_MOESM2_ESM.docx]

**Supplemental File S1. MATERIALS AND METHODS**

**Patient dataset analysis of the I-SPY2-900 trial**

The pretreatment full transcriptome expression data on over 19,000 genes assayed on Agilent 44K data from the I-SPY2-990 clinical trial was accessed from GEO (GSE194040) for the control and pembrolizumab arms. The corresponding clinical information was obtained from the supplemental materials provided by Wolf et al^1^. The data was cleaned using R packages *dplyr*, *tidyr,* and visualized with *ggplot2*. The samples were stratified by treatment arm and differential gene expression between patients exhibiting pathological complete response (pCR) and non-pCR was conducted using the R *limma* package. Additionally, the mRNA data was filtered for ‘*THSD4*’, and the patients were filtered for the pembrolizumab arm of the clinical trial for a total of 69 patients (29 TNBC, 40 ER+). Patients were then binned into subtypes based on receptor expression, i.e. those that were ER+ HER2- were classified as “hormone receptor-positive”, those negative for HR and HER2 were classified as “triple negative”. The *THSD4* expression of those who achieved pCR vs non-pCR was visualized using R packages *ggplot* and *ggpubr*. Significance was determined by Student’s t-test.

**Transcriptome deconvolution of TCGA-BRCA patient dataset**

The CIBERSORTx algorithm^2^ was used to estimate cell proportions of The Cancer Genome Atlas (TCGA) breast invasive carcinoma (BRCA) dataset and METABRIC breast cancer cohort. RPKM counts from the TCGA-BRCA study were acquired using the Illumina HiSeq 2000 RNA sequencing array and were accessed through the BROAD GDAC FireBrowse portal. Gene array data for the METABRIC cohort was accessed through cBioPortal. A signature matrix generated using a comprehensive breast cancer single cell RNA-sequencing dataset^3^. The dataset contained RNA expression for 2697 B cells, 16235 cancer cells, 362 dendritic cells, 2119 endothelial cells, 5888 fibroblast cells, 363 mast cell, 1965 myeloid cells and 14395 T cells. The signature matrix was generated using 200 samples of each cell type. Samples were deconvoluted using B-mode batch correction with 100 permutations. To investigate associations with expression of specific genes on BRCA tumor composition, tumors were stratified into two groups based on high versus low expression of genes of interest. Cell proportion differences across tumor subgroups were analyzed with a non-parametric Mann-Whitney test conducted by the *ggpubr* version 0.5.0 R package.

**Analysis of *THSD4* expression in TCGA BRCA patient datasets**

Tumor-infiltrating lymphocyte fraction (TIL fraction) of total tissue from whole-slide images from The Cancer Genome Atlas (TCGA) archive of H&E stained tumors were determined by Saltz et al.^4^ and accessed from the associated supplemental files. The data was filtered for the breast cancer patients ‘BRCA’ in ‘Study’ column. The clinical data and THSD4 expression for the patients included in the Saltz et al. study were obtained through cBioportal (mRNA vs RSEM)^5,6^ from the Breast Invasive Carcinoma (BRCA TCGA, Cell 2015) dataset. The two datasets were merged based on ‘TCGA Participant Barcode’ then THSD4 expression was paired with TIL fraction for each patient. The patients were subtyped as HER2-enriched if they were positive for HER2 by immunohistochemistry (IHC); HR+ if they were positive for ER or PR and negative for HER2; and triple negative if they were not positive for the three markers by IHC. Once subtyped, the patients were binned into “high”, “medium”, or “low” based on THSD4 expression with approximately a third of the total in each bin. The “high” and “low” groups were compared by T-test.

Kaplan-Meier survival curves were generated based on the subtypes and *THSD4* expression bins generated for the TIL fraction analysis. Survival curve estimates comparing *THSD4* “high” and “low” bins were fit using the *survival* and *survFit* packages, hazard ratios calculated using the *coxph* package, and the curves were visualised using the *ggsurvplot* function from *survminer* in R.

**Local patient tumor cohorts**

All study methodologies with patient tissues conformed to the standards set by the Declaration of Helsinki. The study methodologies were approved by the Nova Scotia Heath (NSH) and IWK Research Ethics Boards. The research ethics board (REB) numbers for the approved protocols 1028015 (NSH REB, “Investigating genetic biomarkers of breast cancer progression and immunosuppression”), 1027711 (NSH REB, “The impact of cancer stem cell and immune cell sub-populations in the efficacy of pembrolizumab treatment in triple-negative breast cancers: a pilot/feasibility study)” and 1023191 (IWK REB, “Breast Biobanking”).

Cohort 1 consists of 15 HR+ breast treatment-naïve tumor samples that were taken at the time of the patients’ primary surgery in 2022 in Nova Scotia, Canada, and were preserved as fresh frozen samples (used for RNA isolation and RNA sequencing (RNAseq)) and formalin fixed paraffin embedded (FFPE) blocks. Analyses of these samples are approved under the above-described REB protocols 1028015 and 1023191. These patient samples were obtained from individuals who had a clear understanding of their samples being accessed for experimental analysis and provided written consent for their samples to be submitted to the NSHA/Dalhousie Biobank. The patient tumor sample details are described in **Supplemental File S3** and was used to assess for correlations between multiplex immunofluorescence staining of the FFPE samples and the RNAseq quantification of THSD4 expression levels.

Cohort 2 consists of 58 treatment-naïve early-stage HR+ and TNBC FFPE patient tumor samples were taken post-surgery from patients who were diagnosed with breast cancer between 2013-2021, from Nova Scotia, Canada. Analyses of these samples are approved under the above-described REB protocols 1028015 and 1023191. For the patient samples dates from 2013-2015, a waiver of consent was granted. The waiver was granted because only a small sample of archived FFPE material post‐diagnosis was used in the study and there was no impact on the patient with respect to treatment choices or prognosis and since many of these patients were treated and diagnosed more than five years ago, we lack relationships with patients and obtaining consent retroactively provided insurmountable difficulties for our group. This Cohort also contains patient samples from the NSHA/Dalhousie Biobank, dating from 2018 and 2021 and hence these patient samples were obtained from individuals who had a clear understanding of their samples being accessed for experimental analysis and provided written consent for their samples to be submitted to the NSHA/Dalhousie Biobank. The patient tumor sample details, along with clinical follow-up date until 2025 are described in **Supplemental File S3**.

Cohort 3 consists of 48 FFPE samples are from patient tumor samples with advanced metastatic TNBC disease from 2022-2024 with clinical follow up until 2025, from Nova Scotia, Canada, under approved REB 1027711. The waiver of consent was granted because only a small sample of archived FFPE material post‐diagnosis was used in the study and there was no impact on the patient with respect to treatment choices or prognosis and since these samples were from palliative patients, in most cases obtaining consent would be impossible. The cohort of patients were subsequently treated with pembrolizumab with chemotherapy or chemotherapy alone depending upon pathological assessment of PD-L1 score of the FFPE tumor sample (score of >10 was the cutoff for eligibility for anti-PD-1 treatment). The PD-L1 assessment was either done on a metastatic lesion if available or if not available, on the primary tumor biopsy core. The treatment of each patient and follow-up clinical data until 2025 (**Supplemental File S**) was used to assess for correlations between survival and the multiplex immunofluorescence staining of the FFPE samples. Notably, eight patients in Cohort 3 did not go on to receive further treatment after PD-L1 assessment and were not included in the survival analysis comparing treatments.

**RNAseq Analysis of Patient Tumor Cohort 1**

Fifteen fresh frozen HR+ patient tumor samples from the treatment naïve cases of Cohort 1 (Supplemental File S3) were homogenized using a Qiagen TissueRuptor II for 30 seconds in buffer RLT Plus (with B-ME) and RNA extracted using the All Prep DNA/RNA Mini kit (Qiagen, Toronto, Canada) as per manufacturer’s protocol. RIN values and DV200% were confirmed with Agilent TapeStation using RNA High Sensitivity. Library preparations were performed with Illumina Stranded Total RNA Prep with Ribo-Zero Plus as per manufacturer’s protocols and RNA libraries are run on the TapeStation to check the library size using the Agilent D1000 tapes.

Raw RNA sequencing data was processed using an analysis pipeline adapted from the Nextflow language-based workflow manager based nfcore pipelines^7–9^. Raw FastQ files were preprocessed for initial QC and adaptors trimmed using fastp (version 0.23.4)^10^. Reads that met the analysis criteria were mapped to the human reference genome (GRCh38) using STAR (version 2.7.10a)^11^. BAMs were sorted and indexed using Samtools (version 1.16.1) and visualized using IGV (version 2.13.0). Duplicate reads were marked using Picard (version 3.1.0)^12,13^. Finally, the FeatureCounts (version 2.0.1). Salmon (version 1.10.1) and Tximport (Bioconductor -tximeta version 1.12.0 and r-base version 4.1.3) were used to perform transcript reconstruction and statistics including TPM count matrix generation^14,15^. All software were used with default settings.

**Opal Multiplex Immunofluorescence Staining of Patient Tumor Cohorts**

The multiplex immunofluorescence protocol was adapted from Nersesian et al.^16^. We used equipment, software, and reagents from Akoya Biosciences unless otherwise indicated. We used antibodies (CD8, CD3, and CD20) validated against cell pellets from primary peripheral blood mononuclear cells (PBMCs) and an antibody for pan-cytokeratin (panCK) validated against tonsil tissue by Nersesian et al.^16^.

Antibodies for CD8 (C8/144; Cat. #GA62361-2) and panCK (AE1/AE3; Cat. #GA05361-2) were acquired from Agilent Dako. CD20 (L26; Cat. #74332S) was purchased from Cell Signalling Technologies. CD3 (LN10; Cat. #MONX11071) was purchased from Monosan. CD45 (CD45-2B11; Cat. #14-9457-82) from eBioscience, and THSD4 (20619-1-AP; Cat. #20619-1-AP) from Proteintech were purchased through Invitrogen.

The steps for staining^16^ were repeated for each antibody in the multiplex panel: CD8 (C8/144, prediluted; Opal 650), CD20 (L26, 1/25; Opal 620), CD3 (LN10, 1/50; Opal 480), THSD4 (20619-1-AP, 1/50; Opal 540). Cohorts 2 and 3 were stained with this panel. The order of antibody staining was optimized empirically. Cohort 1 was stained with various antibody panels. The first panel consisted of panCK (AE1/AE3, 1/50; Opal 650), CD20 (L26, 1/25; Opal 620), CD3 (LN10, 1/50; Opal 520), and CD45 (CD45-2B11, 1/50; Opal 570). The second panel parsed out the T cell populations using CD8 (C8/144, prediluted; Opal 650) and CD3 (LN10, 1/50; Opal 520). The third panel was stained using the Leica Bond autostaining protocol with the validated antibodies and manual Akoya Opal kits. The third panel consisted of panCK (Opal 690), CD8 (Opal 570), CD20 (Opal 650), CD3 (Opal 520), CD45 (Opal 620), and THSD4 (Opal 540).

**Multispectral analysis and automated quantitative pathology**

Imaging was conducted following the protocol by Nersesian et al.^16^. Images were captured at 10x magnification and processed in InForm Tissue Finder Software (Akoya Biosciences) to conduct multispectral analysis (extracting fluorescent signatures) and subsequent quantitative pathology. Tissues were virtually segmented to define regions as tumor epithelia, tumor stroma, vasculature/autofluorescence, and off-core areas. This was followed by cellular segmentation to identify individual cell nuclei based on DAPI staining. CD3, CD8, and CD20 membrane stains were used to aid in cellular segmentation of lymphocytes. Each cell was then assigned a unique cell ID which was used to quantify the antibody signal surrounding the individual nuclei. These antibody signals were used to phenotype cells. CD3+, CD8+, or CD20+ cells were identified as the lymphocyte population. CD3+CD8+ cells were identified as cytotoxic T cells and CD20+ cells were identified as B cells. Tissue THSD4 percent positivity was determined by H-scoring using InForm software. Cells negative for all markers were identified as Other.

For Cohort 1 analysis, cells staining CD45+ but negative for other lymphocyte markers were identified as immune cells/leukocytes. CD45+CD3+CD8- cells were identified as T cells (possibly consisted of CD4+ helper T cells). CD45+CD3+CD8+ as cytotoxic T cells. CD45+CD20+ cells were identified as B cells. Cells staining positive for panCK were identified as epithelial cells. These designations were used as appropriate for the panel.

The exported data was analyzed using the *Phenoptr* and *PhenoptrReports* R packages from Akoya Biosciences. The density of cells in the stroma or epithelium was expressed as defined cell type count per mm^2^ of tissue area. Linear regression models were used for statistical analysis comparing immune cell density to THSD4 positivity score.

**Patient dataset analyses of ICI clinical trials**

Clinical trials investigating the use of ICI in breast cancer with complete gene expression data and clinical data were used to supplement the small sample size of the local Nova Scotian cohorts. THSD4 expression data was compared to clinical response to ICI treatment in three clinical trials. Pre-treatment biopsies were taken from stage IV TNBC patients before treatment with pembrolizumab in a phase Ib clinical trial^17^ and phase II clinical trial^18^. RNA-sequencing (RNAseq) data from an Illumina HiSeq 4000 was accessed from GEO (GSE225078) and the corresponding clinical data was provided by Page et al.^17^. Normalized counts from RNAseq data were accessed from GEO (GSE241876). The corresponding clinical response data was available from Wilkerson et al.^18^.

Normalized RNAseq data from the ‘durvalumab’ arm of the I-SPY2 clinical trial were accessed from GEO (GSE173839) and clinical information was available from Pusztai et al.^19^ Stage II/III HER2-negative breast cancer patients were treated with the anti-PD-L1 durvalumab and PARP inhibitor Olaparib. THSD4 expression from the patients from the ‘durvalumab’ was compared first among those who achieved pathological complete response (pCR) to those who did not, then the analysis was repeated after dividing the groups into HR+ or TNBC subtypes based on the expression of ER or PR.

**Cell culture and reagents**

HEK293T (CVCL 0063) and the triple-negative murine mammary carcinoma 4T1 cells (CRL-2539) and were obtained from the American Type Culture Collection (ATCC, US, VA, Manassas). The hormone receptor-positive TS/A mouse mammary adenocarcinoma cells (SCC177) were obtained from MilliporeSigma (Burlington, MA, United States). 4T1 cells were cultured in RPMI 1640 medium (Invitrogen, Burlington, Canada) supplemented with 10% fetal bovine serum (FBS; Invitrogen) and antibiotic-antimycotic (AA; Invitrogen). HEK293T and TS/A cells were cultured in Dulbecco’s Modified Eagle’s Medium (DMEM; Invitrogen) supplemented with 10% FBS and 1X AA. Cells were grown in a humidified 37°C incubator with 5% CO_2_.

Lentiviral shRNA knockdown clones (**Supplemental Table S1**) were generated using the pLKO.1 vector (Dharmacon, Lafayette, CO, United States) designed by The RNAi Consortium (TRC) TRC-Mm1.0 library as previously described^20^. Briefly, lentivirus was assembled in HEK293T cells using a second-generation packaging system (pMD2.G, pSPAX2). Lentiviral supernatants were collected and filtered (0.2μm) prior to being applied to 4T1 and TS/A cells. Clones were selected by administering 4.5 or 5.5μg/mL puromycin for 48-72 hours to 4T1 and TS/A cells, respectively, and subsequently maintained in 1.0μg/mL puromycin (Invitrogen).

**Reverse transcriptase-quantitative polymerase chain reaction (RT-qPCR)**

Knockdown of *Thsd4* in murine mammary carcinoma cells was determined by RT-qPCR using two different primer sets. Cells were collected in Trizol (Invitrogen), and RNA was purified using a PureLink RNA kit with DNase treatment (Thermo Fisher Scientific, Mississauga, ON, Canada) following the manufacturer’s instructions. Equal amounts of purified RNA were then reverse transcribed to cDNA using iScript (Bio-Rad, Mississauga, ON, Canada) as per the manufacturer’s instructions. Diluted cDNA was used in RT-qPCR reactions with gene-specific primers and SsoAdvanced Universal SYBR Supermix (Bio-Rad) as per the manufacturer’s instructions with a CFX96 or CFX384 Touch Real-Time PCR Detection System (Bio-Rad). Standard curves were generated for each primer set and primer efficiencies were incorporated into the CFX Manager software (Bio-Rad). Relative expression for decitabine inducible genes was quantified using the ΔΔct method of the CFX Manager Software (Bio-Rad), where gene-of-interest quantification was normalized to reference genes mouse *Gapdh* and *B2m* and then made relative to the shRNA control cDNA sample.

**Mouse Studies**

All animal studies have been conducted in accordance with the ethical standards and according to the Declaration of Helsinki and the Canadian Council on Animal Care (CCAC) standards and performed under the protocol #22-014 approved by the Dalhousie University Committee on Laboratory Animals. Seven- to eight-week-old female Balb/c mice from Charles River Laboratories (Senneville, QC) were used for all experiments in this study. Mice were anaesthetized with isoflurane gas in an induction chamber for implantation of breast cancer cells and euthanized at endpoint with isoflurane gas and carbon dioxide gas. Tumor volume was quantified as (mm^3^, [length x width x height] / 2).

1.0 x10^4^ 4T1 or TS/A cells with stable *Thsd4* knockdown were orthotopically injected into the lower mammary fat pad of 8–9-week-old female Balb/c mice. Mice were treated with rat IgG2a isotype control (Bio X Cell, Lebanon, NH, United States) or anti-mouse PD-1 (CD279) (Bio X Cell). After the development of palpable tumors (day 8), mice were divided into isotype control or anti-PD-1 groups. Mice received intraperitoneal injections every other day of 100uL of 2.0mg/mL antibody diluted in PBS until humane endpoint. Mice injected with 4T1 cells (n=40, 10/group) were euthanized on day 18, mice injected with TS/A cells (n=60, 15/group) were euthanized on day 21. Tumors were excised and weighed prior to mincing for RNA extraction, formalin fixation for histological analysis, or processing for flow cytometry as described below.

**Flow cytometry analysis of tumors for lymphocytes**

Excised tumors from TS/A and 4T1 tumors were minced and strained through a 40µm filter to create a single-cell suspension. All cell washes were completed with flow cytometry running buffer (PBS-EDTA with 1% FBS; FACS buffer). Red blood cell lysis was performed using ACK buffer for 5 min at room temperature. 1-2 x 10^6^ cells were collected and stained with viability dye (APC eFluor780, Thermo-eBiosciences, #65-0865-18) for 15 min at room temperature (RT). Cells were washed then incubated with an anti-CD16/32 FC-block for 20 min at 4°C. Cells were washed then stained with anti-mouse CD45 (CD45 - BV786, BD Biosciences, #564225), CD3 (CD3 - BV421, BioLegend, #100341), CD8 (CD8 - PerCpCy5.5, BioLegend, #100734), CD4 (CD4 – FITC, BioLegend, #100510), CD19 (CD19 – APC, BioLegend, #115512), PD-1 (PD-1 – BV605, BioLegend, #135220), PD-L1 (PD-L1 – PE, BioLegend, #124308), and for 30 min at 4°C with 0.5µL of each antibody per sample. Cells were washed, fixed with 4% formaldehyde for 15 min at RT, and resuspended in FACS buf fer prior to analysis. The analysis was performed on the Fortessa flow cytometer (BD Biosciences, Franklin Lakes, NJ, United States) and analyzed using FCS express (De Novo Software, Pasadena, Ca, United States).

**Histological analysis of collagen fibers in mouse tumors by picrosirius red staining**

Harvested tumor tissues were fixed in 10% acetate-buffered formalin for 24 hours, then rinsed and stored in 70% ethanol. Tissues were dehydrated, cleared with xylene, and embedded in paraffin. Five of the tumors from each group were randomly chosen and 4µm section cut stained by picrosirius red as per standard protocol^21^. Tumors were imaged using a Nikon Digital Sight 10 camera on a Nikon eclipse Ni microscope and processed using NIS-Elements D software (v5.41.02). Three representative images were taken for each tumor, then analysed using the protocol and ImageJ plugin from Vennin et al.^22^, which calculates the polarized signal from each image, then further classifies based on Red-Orange, Yellow, or Green coloured fibers. The mean and standard deviation for the three colour channels from each tumor was calculated then grouped based on intervention.

**Statistical analyses**

Statistical analyses were performed in the GraphPad Prism software (GraphPad Software, San Diego, CA, USA), unless otherwise mentioned. In all cases where three or more groups are compared, a one-way ANOVA was performed (with multiple comparisons post-test as indicated in the figure legend). Comparisons between two groups were done using a two-tailed Student’s t-test or Mann-Whitney test for non-normally distributed samples. Significant p values are indicated as follows in the figures: p < 0.05 = *, p < 0.01 = **, p < 0.001 = ***, p < 0.0001 = ****. We calculated the sample size needed to detect a significant difference in anti-THSD4 staining between two groups of late-stage metastatic TNBC patients treated with pembrolizumab, categorized by worse versus better survival based on median survival analysis^23,24^. Using the preliminary group means and standard deviations, we computed Cohen’s *d* effect size with the pooled standard deviation. A two-sided independent samples t-test with α = 0.05 and 80% power was used to estimate the required number of patients per group.

**References**

1. Wolf, D. M. *et al.* Redefining breast cancer subtypes to guide treatment prioritization and maximize response: Predictive biomarkers across 10 cancer therapies. *Cancer Cell* **40**, 609-623.e6 (2022).

2. Newman, A. M. *et al.* Determining cell type abundance and expression from bulk tissues with digital cytometry. *Nat. Biotechnol. 2019 377* **37**, 773–782 (2019).

3. Qian, J. *et al.* A pan-cancer blueprint of the heterogeneous tumor microenvironment revealed by single-cell profiling. *Cell Res. 2020 309* **30**, 745–762 (2020).

4. Saltz, J. *et al.* Spatial Organization and Molecular Correlation of Tumor-Infiltrating Lymphocytes Using Deep Learning on Pathology Images. *Cell Rep.* **23**, 181-193.e7 (2018).

5. Cerami, E. *et al.* The cBio cancer genomics portal: an open platform for exploring multidimensional cancer genomics data. *Cancer Discov.* **2**, 401–404 (2012).

6. Gao, J. *et al.* Integrative analysis of complex cancer genomics and clinical profiles using the cBioPortal. *Sci. Signal.* **6**, (2013).

7. Ewels, P. A. *et al.* The nf-core framework for community-curated bioinformatics pipelines. *Nat. Biotechnol. 2020 383* **38**, 276–278 (2020).

8. Ewels, P., Magnusson, M., Lundin, S. & Käller, M. MultiQC: summarize analysis results for multiple tools and samples in a single report. *Bioinformatics* **32**, 3047 (2016).

9. DI Tommaso, P. *et al.* Nextflow enables reproducible computational workflows. *Nat. Biotechnol. 2017 354* **35**, 316–319 (2017).

10. Chen, S., Zhou, Y., Chen, Y. & Gu, J. fastp: an ultra-fast all-in-one FASTQ preprocessor. *Bioinformatics* **34**, i884–i890 (2018).

11. Dobin, A. *et al.* STAR: ultrafast universal RNA-seq aligner. *Bioinformatics* **29**, 15 (2012).

12. Li, H. *et al.* The Sequence Alignment/Map format and SAMtools. *Bioinformatics* **25**, 2078–2079 (2009).

13. Robinson, J. T. *et al.* Integrative genomics viewer. *Nat. Biotechnol. 2011 291* **29**, 24–26 (2011).

14. Patro, R., Duggal, G., Love, M. I., Irizarry, R. A. & Kingsford, C. Salmon provides fast and bias-aware quantification of transcript expression. *Nat. Methods 2017 144* **14**, 417–419 (2017).

15. Soneson, C., Love, M. I. & Robinson, M. D. Differential analyses for RNA-seq: Transcript-level estimates improve gene-level inferences. *F1000Research* **4**, (2016).

16. Nersesian, S. *et al.* Improved overall survival in patients with high-grade serous ovarian cancer is associated with CD16a+ immunologic neighborhoods containing NK cells, T cells and macrophages. *Front. Immunol.* **14**, 1307873 (2023).

17. Page, D. B. *et al.* A phase Ib trial of pembrolizumab plus paclitaxel or flat-dose capecitabine in 1st/2nd line metastatic triple-negative breast cancer. *npj Breast Cancer 2023 91* **9**, 1–13 (2023).

18. Wilkerson, A. D. *et al.* Phase II Clinical Trial of Pembrolizumab and Chemotherapy Reveals Distinct Transcriptomic Profiles by Radiologic Response in Metastatic Triple-Negative Breast Cancer. *Clin. Cancer Res.* **30**, 82–93 (2024).

19. Pusztai, L. *et al.* Durvalumab with olaparib and paclitaxel for high-risk HER2-negative stage II/III breast cancer: Results from the adaptively randomized I-SPY2 trial. *Cancer Cell* **39**, 989-998.e5 (2021).

20. Stewart, S. A. *et al.* Lentivirus-delivered stable gene silencing by RNAi in primary cells. *RNA* **9**, 493 (2003).

21. Rittié, L. Method for picrosirius red-polarization detection of collagen fibers in tissue sections. *Methods Mol. Biol.* **1627**, 395–407 (2017).

22. Vennin, C. *et al.* CAF hierarchy driven by pancreatic cancer cell p53-status creates a pro-metastatic and chemoresistant environment via perlecan. *Nat. Commun.* **10**, (2019).

23. Althubaiti, A. Sample size determination: A practical guide for health researchers. *J. Gen. Fam. Med.* **24**, 72 (2022).

24. Wang, X. & Ji, X. Sample Size Estimation in Clinical Research: From Randomized Controlled Trials to Observational Studies. *Chest* **158**, S12–S20 (2020).
